# Continuity and Discontinuity of Sport and Exercise Type During the COVID-19 Pandemic. An Exploratory Study of Effects on Mood

**DOI:** 10.3389/fpsyg.2021.622876

**Published:** 2021-02-12

**Authors:** Noora J. Ronkainen, Arto J. Pesola, Olli Tikkanen, Ralf Brand

**Affiliations:** ^1^Department of Psychology, University of Jyväskylä, Jyväskylä, Finland; ^2^Active Life Lab, South-Eastern Finland University of Applied Sciences, Mikkeli, Finland; ^3^Physical Activity Researcher Podcast, Jyväskylä, Finland; ^4^Sport and Exercise Psychology, University of Potsdam, Potsdam, Germany; ^5^Department of Kinesiology, Iowa State University, Ames, IA, United States

**Keywords:** exercise behavior, being-in-the-world, lockdown, worldmaking, profile of mood states, existential philosophy, affect

## Abstract

Involvement in sport and exercise not only provides participants with health benefits but can be an important aspect of living a meaningful life. The COVID-19 pandemic and the temporary cessation of public life in March/April/May 2020 came with restrictions, which probably also made it difficult, if not impossible, to participate in certain types of sport or exercise. Following the philosophical position that different types of sport and exercise offer different ways of “relating to the world,” this study explored (dis)continuity in the type of sport and exercise people practiced during the pandemic-related lockdown, and possible effects on mood. Data from a survey of 601 adult exercisers, collected shortly after the COVID-19 outbreak in Finland, were analyzed. Approximately one third (35%) of the participants changed their “worldmaking” and shifted to “I–Nature”-type activities. We observed worse mood during the pandemic in those who shifted from “I–Me,” compared to those who had preferred the “I–Nature” relation already before the pandemic and thus experienced continuity. The clouded mood of those experiencing discontinuity may be the result of a temporary loss of “feeling at home” in their new exercise life-world. However, further empirical investigation must follow, because the observed effect sizes were small.

## Introduction

The governmental lockdowns (i.e., restrictions of travel and closures of schools, workplaces, exercise/sport facilities instituted as a safety measure) following the Coronavirus outbreak from early 2020 onwards imposed drastic changes in people's daily lives, with possible physical, social, and psychological consequences. Although the lockdowns prevented the explosive spread of the virus in many countries, there were side effects. Commentaries have warned that imposing a sudden and strict lockdown can bring a radical discontinuity to people's routines and lifestyle, including participation in sport and exercise (Begović, 2020; Hammami et al., 2020). Whereas some studies have reported a decrease in adults' physical activity since the onset of the pandemic, for example in the US (Meyer et al., 2020), in Australia (Stanton et al., 2020), and in an international sample from 14 countries (Wilke et al., 2020), different studies have observed maintenance or even increases in physical activity during the pandemic. Based on an analysis of Google Trends in the UK, the USA, and Australia, Ding et al. (2020) found that the public interest in exercise surged quickly following the first lockdowns, and other studies have shown behavioral changes toward increased physical activity and exercise. For example, Smith et al. (2020) showed that during the first lockdown in early 2020 more adults (75%) in the UK have met the physical activity guidelines, compared to the situation before the lockdown (between 58 and 66%). According to an international study with more than 13.000 participants from 18 countries worldwide, 31.9% reported having started exercising more frequently during the lockdown, whereas 44.2% reported no change, and only 23.7% reported a decrease of usual exercise frequency (Brand et al., 2020). At first glance, some of these results seem to be contradictory. This impression is put into perspective, however, as soon as one takes into account that the various studies have in fact measured different aspects of physical activity and exercise (e.g., cumulated moderate and vigorous physical activity minutes per day, frequency and duration of walking, or exercise session frequency per week).

Scholars have noted that potential negative psychological effects of pandemic-related lockdown may include post-traumatic stress symptoms, anxiety, depression, anger, and confusion (Qiu et al., 2020). However, on the other hand, positive responses such as trust, collective solidarity, and altruism have also been seen (Barkur et al., 2020; Sun et al., 2020). Emerging studies have shown an association between reduced amounts of physical activity during the pandemic and symptoms of stress and depression (Meyer et al., 2020; Stanton et al., 2020). Brand et al.'s (2020) findings from the large international study concluded that those who exercised most during the lockdown reported better mood.

Until now, most of the studies have focused on changes in amounts of physical activity or exercise, and not whether people have changed the type of activities. In this study, we used the Finnish subsample of the large international study conducted by Brand et al. (2020) to explore whether maintaining or having to change one's usual form of exercise in the wake of a sudden lockdown (due to the unexpected closure of exercise facilities and restrictions of gatherings) would impact people psychologically. We focus on the effect of (dis)continuity, i.e., the question of whether the continuation or sudden change in exercise or sport type is related to general mood. Our approach is theoretically based on the existential philosophical conceptualizations of mood (Ratcliffe, 2013; Freeman, 2014) and the idea of sport and exercise as a way of worldmaking (Breivik, 2020). If we change–or are forced to change–the way we engage with the world through our sport or exercise, this different way of being-in-the-world might also have an impact on mood.

### Exercise and Mood

Mood is a much-studied and often measured concept in sport and exercise psychology, but the elusive and complex foundations of moods' philosophical meanings are rarely explained. In the following, these two different analytical approaches (psychological, philosophical) to mood are briefly described.

Conceptualizations of mood are similar in the parent discipline of psychology and sport and exercise psychology (e.g., Ekkekakis, 2013). While emotion is typically short, intense, and “about something” (i.e., emotions are always a response to a specific stimulus), mood has a longer duration, is of lower intensity, and does not need to be about anything in particular (Ekkekakis, 2013). Moods are primarily seen as indicators of psychological disturbance or well-being, and almost always as a reaction of the individual to the world outside.

On the other hand, in philosophy, there is a more existentialist perspective to the understanding of mood and its constitutive role in human lives. From this perspective experiencing moods is not only a reaction to encounters of our daily going-about, but rather part of the individual's constructive access to the world: Mood is the basic mode through which the world discloses itself to us, and how we experience the world (Freeman, 2014). Drawing on Heidegger, Ratcliffe (2013) suggested that “moods constitute how we *find ourselves in the world*” (p. 157) or “*belong to a world*” (p. 158). Moods are neither simple inner psychological states, nor “out there” in the world. They are something “in-between,” giving us “a sense of being part of a world that is pre-subjective and pre-objective” (Ratcliffe, 2013, p. 157).

According to this philosophical perspective, moods concern the way we are practically immersed in our world, and what we find important or irrelevant. These matterings are shaped by cultural webs of significance, such as how sport and exercise are generally understood in our cultural environment. Action and world are interwoven by moods. The COVID-19 pandemic has likely changed the existential background of our lives, which in turn is likely to affect the way we are and feel in this “new world.”

Despite the different ways of how mood has been conceptualized in philosophy and how measures of mood have been used in psychological research, the two perspectives may also be related to each other. Empirical studies showed that aerobic as well as resistance exercise may both have acute and long-term positive effects on mood (Berger and Motl, 2000; Chase and Hutchinson, 2015). It has been emphasized, however, that some individuals may experience little or no psychological benefits due to situational, personal, or activity-related other factors (Rocheleau et al., 2004). For example, individuals who do not exercise regularly might experience only limited improvement in their mood from a bout of exercise compared to trained individuals (Hoffman and Hoffman, 2008). Findings like these highlighted that mood is connected to our already established relationship with exercise and is not a simple, automatic response to physical exertion.

This is further supported by findings from the international study of which this study is a part (Brand et al., 2020). Exercise frequency before the COVID-related lockdown was a protective factor for mood during it, in the way that those who only started during the lockdown and exercised infrequently did not report better mood. Expectations about the outcomes from exercise, stemming from previous personal experiences and cultural frameworks of meaning surrounding exercise, seem to play a role in how an exercise session impacts mood (Anderson and Brice, 2011; Mothes et al., 2017). In contrast to popular beliefs, the “feel good” effect of exercise is not automatic, but there can be marked interindividual differences in how we experience exercise and how it impacts our well-being (Ekkekakis and Brand, 2019).

### Does the Type of Exercise Matter?

Earlier psychological studies already sought to investigate whether different types of exercise, typically aerobic or resistance training, are differentially related to mood outcomes (e.g., Chase and Hutchinson, 2015). In contrast to these earlier studies, we choose the more philosophically-informed theoretical perspective. We are interested in whether and how different ways of exercising might offer different ways of relating to the world or “worldmaking” (Breivik, 2020).

Breivik focused on participation in sports as a way of exploring the environing world and one's possibilities. He proposed that different sporting activities (e.g., running on the track, playing football, boxing, and kayaking) operate on different primary, ontological relationships that are constitutive of these activities. The relations were termed “I–Me,” “I–You,” “I–Society,” and “I–Nature.” According to Breivik, they are the basis for involvement in sporting activities and contribute to the individual's “worldmaking.” We believe that this framework can be applied to noncompetitive physical exercise as well.

The I–Me relation refers to our own relationship with ourselves and how we find out about our capabilities and limitations through involvement in sport and exercise. Related to competitive sports, this comes with questions like: How fast can I run? How far can I throw? How much can I lift? In the exercising context, respective questions may be: Can I increase my physical fitness? Can I shape my body? In the I-Me relation, the subject remains his or her own reference point. The focus is on exploring and extending one's boundaries and possibilities, while the presence of others is not necessary for the activity to be pursued. Most typically, this relation ideally manifests in individual sports, such as athletics, gymnastics, or weightlifting; however, it is also relevant to non-competitive activities, and even group exercise classes, when the activity is meant to relate to one's personal concerns first of all (e.g., health, body shape, strength, endurance; and not in relation to other participants).

The I–You relation refers to testing ourselves against others in contest or combat, that is, “encounter” sports. Here, the question is about my capacities in relation to your capacities: My cleverness, skill, and strength are measured against yours. The presence of the opponent is a necessity for the activity. Breivik (2020) mentions various martial arts, table tennis, squash, and badminton as examples. Importantly, the nature of the encounter is of rivalry and conflict (and therefore sports dance is not included in this dimension). One person wins, the other loses. However, both parties may still gain benefits from involvement in these activities, and they may be carried out in a friendly manner (e.g., playing recreational tennis).

The I–Society relation concerns our relation to the collective other: being a team member, belonging to a group. This relation manifests in team sports that offer us possibilities for cooperation (but also conflict) and that we may achieve more together than we can achieve alone. For these activities, the presence of the group or team is a necessity. The lockdowns following the COVID-19 outbreak has likely brought the heaviest disruption for those preferring this type of relating to the world, given that group and team sports exercising and training, as well as competitions, ceased in many countries worldwide.

Finally, I–Nature refers to those activities where human beings explore and experiment with their relationship to the natural world. In contrast to a stadium, gym or sports hall, nature is not a stable and predictable arena for testing one's capacities; it can be uncontrollable and perhaps takes the moving person by surprise. The waves in the sea (for the surfer), snow conditions (for the snowboarder), rain, wind, obstacles, or animals on the path (for the runner) present unanticipated encounters for the moving person. Nature is alive and can be acting on us; but always incidentally, and not intentionally as opponents as in the I–You and I–Society types would.

Breivik (2020) noted that this fourfold framework presents ideal types that cannot always be neatly separated from each other in reality. For example, cycling is an activity that may be experienced by the individual as an I–Me relation (as an individual sport; e.g., a time trial on track), an I–Society relation (team sport; e.g., the Tour de France), or an I–Nature relation (outdoor sport; e.g., mountain biking), or as a combination of all those relations. In addition, not all activities that are carried out in nature *necessarily* prioritize the I–Nature relation. Citing Howe (2012), Breivik notes that it is possible to approach activities in a “‘nature–instrumental” attitude, where nature is seen simply as the platform for one's self-project (e.g., improving fitness or winning a fell running competition). Approaching an activity with a “nature-directed” attitude, where we place value on connecting and resonating with nature, is different from that. Despite these complications, Breivik (2020) argued that the fourfold framework of I–Me, I–You, I–Society, and I–Nature can help identify what is *necessary* and *sufficient* for different sport and exercise activities to be realized. We believe that it can well expand our basic understanding of how we *are* in the sporting world.

### Situating the Study: The Nordic and Finnish Context

The Nordic region is characterized by strong physical activity, “sport for all,” and outdoor life cultures (Bergsgard et al., 2019). In addition to volunteer-based sport clubs, outdoor physical activity has been an important part of the Nordic movement culture heritage and is enabled by the “everyman's right” of access to both public and private forests (Neuvonen et al., 2018). Finland has a low population density and cities are relatively green compared to many other European cities. Most Finnish people engage in physical activity in outdoor spaces, and it has been found that having access to green spaces improved self-rated health through an increase in participation in outdoor physical activity (Pietilä et al., 2015). However, although Finland ranks high in international comparisons and the level of leisure-time physical activity has increased, more than half of the adult population still does not meet the official recommendations for physical activity (Wennman et al., 2019). Wennman et al. (2019) also reported that young and highly educated adults are more physically active than older and less-educated adults.

A national survey in 2009–2010 (Suomen Kuntoliikuntaliitto, 2010) indicated that most of the top-10 forms of exercise and sport activities (in terms of participation rate) among Finnish adults are self-organized and can be undertaken alone. Thus, in Breivik's (2020) terms, they can be categorized into I–Me or I–Nature activities. Walking (1,790,000 participants), cycling (845,000 participants), and weight training at a gym (713,000) were the three most popular types of exercise in Finnish adults, with cross-country skiing, jogging/running, swimming, gymnastics (including aerobic), and Nordic walking also included in the top-10 activities. In addition to these, only one I–You (badminton) and one I–Society (floorball) activity were included in the top-10. This said, 72% reported that they took part in two or more different types of activities. However, some activities are seasonal (e.g., skiing or skating in the winter, kayaking or orienteering in the summer), and for athletes, their second type of activity (e.g., weight training) might rather represent a necessary means to improve performance in their primary sport.

### This Study

While the main objective of Breivik's (2020) philosophical discourse was not to categorize different sport and exercise activities into the proposed four basic “relations to the world,” he mentioned that the fourfold framework may be applied in that way in other studies. This is what we tried to do here. Of course, we fully recognize the empirical complications that come with this approach in terms of classifying the different activities, and the challenge of inferring people's intentions from survey data (i.e., whether participants adopted, for example, a nature-instrumental or nature-directed attitude toward their activities). However, since our focus is on continuity and discontinuity in people's behavioral preferences, and not on the analysis of subjective experiences, we find the fourfold framework useful in giving us indication of whether the mode of participants' exercise or sport activities has meaningfully changed in response to the COVID-19-related lockdowns.

We used the Finnish subsample from a larger international study (Brand et al., 2020) as data for our explorations. Following Breivik's (2020) fourfold framework, we categorized the participants' preferred type of relating to the world through sport or exercise, before and during the pandemic. We expected that as a result of the lockdown, many participants who had been involved in indoor exercise and/or group activities before (e.g., an I–Me relation, such as visiting gyms, I–You relations, such as doing martial arts, or I–Society relations, such as playing football), were forced to change their primary mode of engagement. Assuming that such shifting would affect our participants' worldmaking, we explored whether these shifts would indeed affect their moods as measured with a psychological scale. Given the explorative nature of this approach, we did not set up hypotheses about the direction of possible changes.

## Materials and Methods

The data we used for the present study were collected as part of a worldwide survey with more than 16,000 participants from all over the world, which was conducted in a joint effort of the International Research Group (IRG) on COVID and exercise (Brand et al., 2020). All members of IRG are listed, and the exact methodology of the study, is described in detail in the International Study Report. The Finnish data we are investigating here were collected between April 8, 2020, and May 31, 2020.

### Participants

We analyzed the data of 601 adults aged 18–73 years (227 men, 367 women, 7 other; mean age of the total sample was 41.8 ± 11.4 years) residing in Finland. Most of them had a university master's degree or had received higher education (426), were working full time (447), and reported having a medium or high income (497). An about equal number of participants reported living in an urban (245 participants) or a suburban region (267). Fewer participants reported residing in a rural region (88).

### Data Collection and Ethics

As part of the international study, the Unipark™ web-based survey-software was used for data collection, with Finnish participants being recruited by convenience sampling. We advertised the study and contacted potential participants via social media platforms (Twitter, LinkedIn, Facebook), and used the authors' private and professional networks (e.g., email lists). We followed the General Data Protection Regulations (EU) and the American Psychological Association (APA) Ethical Guidelines for Research, and participants provided informed consent prior to the study. The questionnaire was anonymous, and it was also possible to skip questions or stop participating at any point.

### Study Variables

#### Exercise

Exercise was defined for the participants of this study in the way that “Exercise in a broader sense includes all movement activities that you choose to do as “your exercise.” This includes, for example, purposefully undertaken walks as well as fitness training, workouts at home, football, swimming, and others.” Participants were also informed that any physical activity that was part of their occupation should not be included when answering this question (unless they were a professional fitness coach or had a similar profession).

The two questions asked were: “How often did you exercise in the weeks before COVID-19?” and “How often have you exercised lately (during COVID-19)?” Possible answers were “never” or “less than once a week” (these two categories were collapsed for statistical analysis), and then from “1 day per week” in single-day steps up to “every day.” Those reporting exercise were asked to type in their answer to the question “What type of exercise did you complete on most of these days?”

Usual exercise intensities before and during the pandemic were asked about with the question “What would you say the intensity of this exercise was each time you did it?” (low, moderate, or high intensity). With regard to session durations, we asked whether these exercise sessions had been “on average shorter or longer than before COVID-19?” Possible answers to this question were “shorter,” “longer,” or “they were of about the same duration.”

#### Mood

Selected items from the Profile of Mood Scale (POMS) (McNair et al., 1971) were used for measuring our study participants' general mood state during the lockdown. The 16-item version of POMS used in the present study is based on a German short screening version (Petrowski et al., 2020), which has been psychometrically tested in a large, representative German sample (list of items available upon request from the corresponding author of this article). German items were matched with the English items as accurately as possible by Brand et al. (2020), and then translated to Finnish by authors 1–3 of the present study.

The POMS presents a list of adjectives that describe experiential states people can have (e.g., “fatigued” and “active”). In our study, we asked the participants to report how they felt “in the last few days, during COVID-19.” They rated each item by indicating whether they experienced the respective feeling “not at all,” “a little,” “moderately,” “quite a lot,” or “extremely” now and/or in the past few days.

The 16 items of the German POMS-16 version can be assigned to the four subscales of depression/anxiety, vigor, fatigue, and irritability. However, for our Finnish translation (which was created from the original German version that was translated into English by Brand et al., 2020) only the more robust POMS-16 total score was used (as any other procedure would have required closer psychometric testing with separate samples, which were not available to us). For the analyses presented here, all items were coded such that higher POMS score indicates better mood. The scale reliability achieved in our sample for the total score was very good (internal consistency; Cronbach's α = 0.88).

#### Personal Information

Demographic questions included age, gender, education, and current place of residence. Also, we asked about the presence of COVID-19 symptoms or a positive diagnosis to exclude these individuals from the statistical analyses.

### Data Coding: Type of Exercise

Participants entered their primary type of exercise as free text. The first author of this article manually coded and interpreted the answers according to Breivik's four relations I–Me, I–You, I–Society, and I–Nature. For example, participants reported gym, crossfit, and pilates, which were coded as I–Me relations. Examples for I–You are badminton, tennis, and kickboxing; for I–Society, football, volleyball, and ice hockey; for I–Nature, jogging/running, orienteering, and hiking. The coding was reviewed by the third author, and ambiguous cases were discussed and subsequently resolved. The full list of exercise types and how they were coded is available in Table 1 of the Supplementary Material to this article.

We recognized that the distinction between I–Me and I–Nature in particular is ambiguous and requires interpretation. As an example, a jogger in outdoor spaces could be focused on the I–Me relation (i.e., jog solely to improve fitness) or I–Nature relation (i.e., jog to enjoy being outdoors), or conceivably often values both (being in natural environments *and* improving fitness). Finnish people in cities also typically have access to green spaces for exercising (Pietilä et al., 2015), and nature areas have been reported to be the most common favorite places for Finnish adults (Korpela et al., 2010). We, therefore, classified walking and jogging/running as I–Nature activities, which was further justified by research indicating that being in nature seems to act *on us* regardless of our intentions e.g., the proximity of green spaces can provide a buffer for stressful life events; Van den Berg et al. (2010). This highlights that we are not merely producing our experiences by intentional activity, but we respond to the world and what is “disclosed” to us (Breivik, 2020).

### Statistical Explorations

All variables were checked as to whether they met the requirements for statistical testing (e.g., normal distribution with Shapiro-Wilk tests and by visual inspection of QQ- and density plots, homogeneity of variance with Levene's test). If violated, non-parametric tests were used for further analysis.

The proportions of how many participants remained or shifted from one relation to another were inspected by cross-tabling this information. Main flows were inspected with a Sankey diagram. From this analysis, change groups (e.g., from I–Me before the lockdown to I–Nature during the lockdown), and maintenance groups (e.g., I–Nature before and also during the lockdown) were formed, which we will refer to as continuity/discontinuity patterns.

We then inspected the continuity/discontinuity patterns with regard to exercise characteristics. In order to explore differences in exercise frequency (days per week) before and during the lockdown, a Wilcoxon signed ranked test for paired samples (non-parametric) was calculated. The same test was used to explore changes in exercise intensity. A Kruskal-Wallis test (non-parametric) was employed for exploring differences in “exercise duration,” as this variable was measured as a difference variable in the survey (no separated pre–post measures available).

Possible differences in mood between participants from the continuity/discontinuity patterns were explored by ANOVA with “mood” as the dependent variable, and “exercise continuity/discontinuity pattern” as a factor. Univariate outliers were evaluated with boxplot methods. All detected outliers represent psychologically meaningful POMS scores, and deleting them did not change our main results. Therefore, we decided not to remove them from the analyzed data. Tukey *post hoc* tests were used for pairwise comparisons to further explore differences between continuity and discontinuity patterns, for significant ANOVAs. Additional ANCOVA's were calculated to check whether possible results are influenced by exercise frequency, session duration, or intensity.

## Results

### Changes Between Exercise Type Categories Following the Lockdown

**Figure 1:**
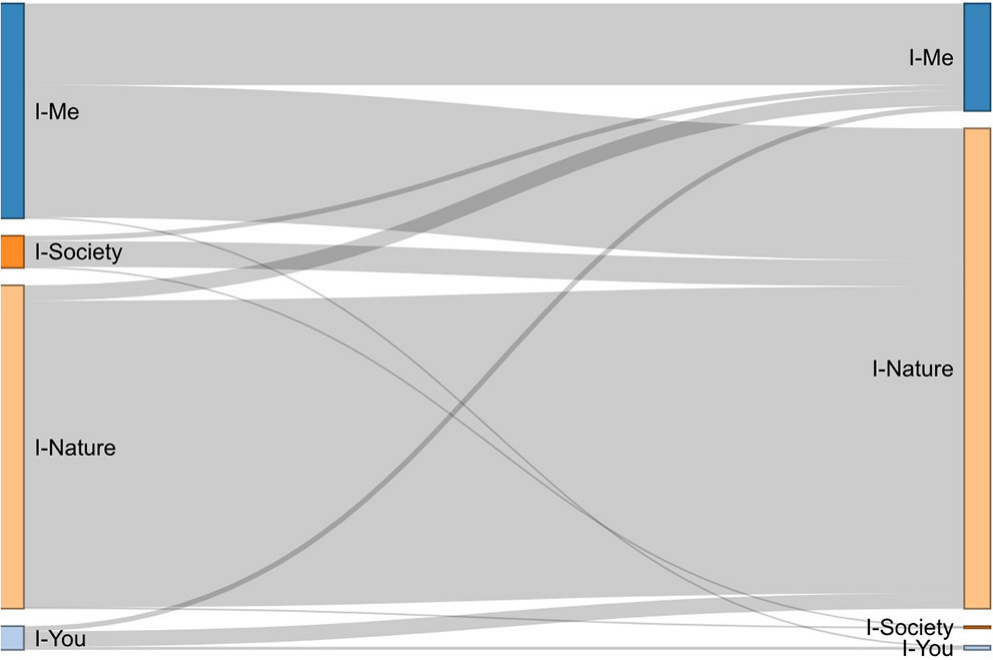
Participant flow between exercise type categories (“Ways of relating to the world”) before and during the lockdown.

Table 1 shows the distribution of participants in the four groups of exercise types (i.e., relations to the world) before and during the lockdown. Figure 1 illustrates the flows from and to the four types. Most of the participants were initially and remained in the I–Nature group during the lockdown (I–Nature continuity pattern: 297 participants). The second most typical pattern was a change from I–Me to I–Nature, that is a shift from indoor exercise (e.g., gym training) to exercising or doing sports in natural environments (I–Me/I–Nature discontinuity pattern: 128 participants). A smaller number of participants remained in I–Me (I–Me continuity pattern: 79 participants) or changed from I–Nature to I–Me (I–Nature/I–Me discontinuity pattern: 15 participants).

**Table 1:** Shifts between exercise type categories (“Ways of Relating to the World”).

	**Exercise type category during the pandemic**				
**Exercise type category before the pandemic**	**I–Me**	**I–You**	**I–Society**	**I–Nature**	**Total**
I-Me	38% (79)	0% (1)	0% (0)	62% (128)	100% (208)
I-You	22% (5)	13% (3)	0% (0)	65% (15)	100% (23)
I-Society	16% (5)	0% (0)	3% (1)	81% (25)	100% (31)
I-Nature	5% (15)	0% (0)	0% (1)	95% (297)	100% (313)
Total	18% (104)	1% (4)	0% (2)	81% (465)	100% (575)

The majority of our study participants were exercising (before and after) in the I–Me and the I–Nature type of activities. Due to the comparatively small proportions of participants in other relations, we decided to focus our statistical explorations on those within the I–Me and I–Nature relations.

### Differences Between Continuity/Discontinuity Patterns in Exercise Intensity, Frequency, and Duration

**Figure 2:**
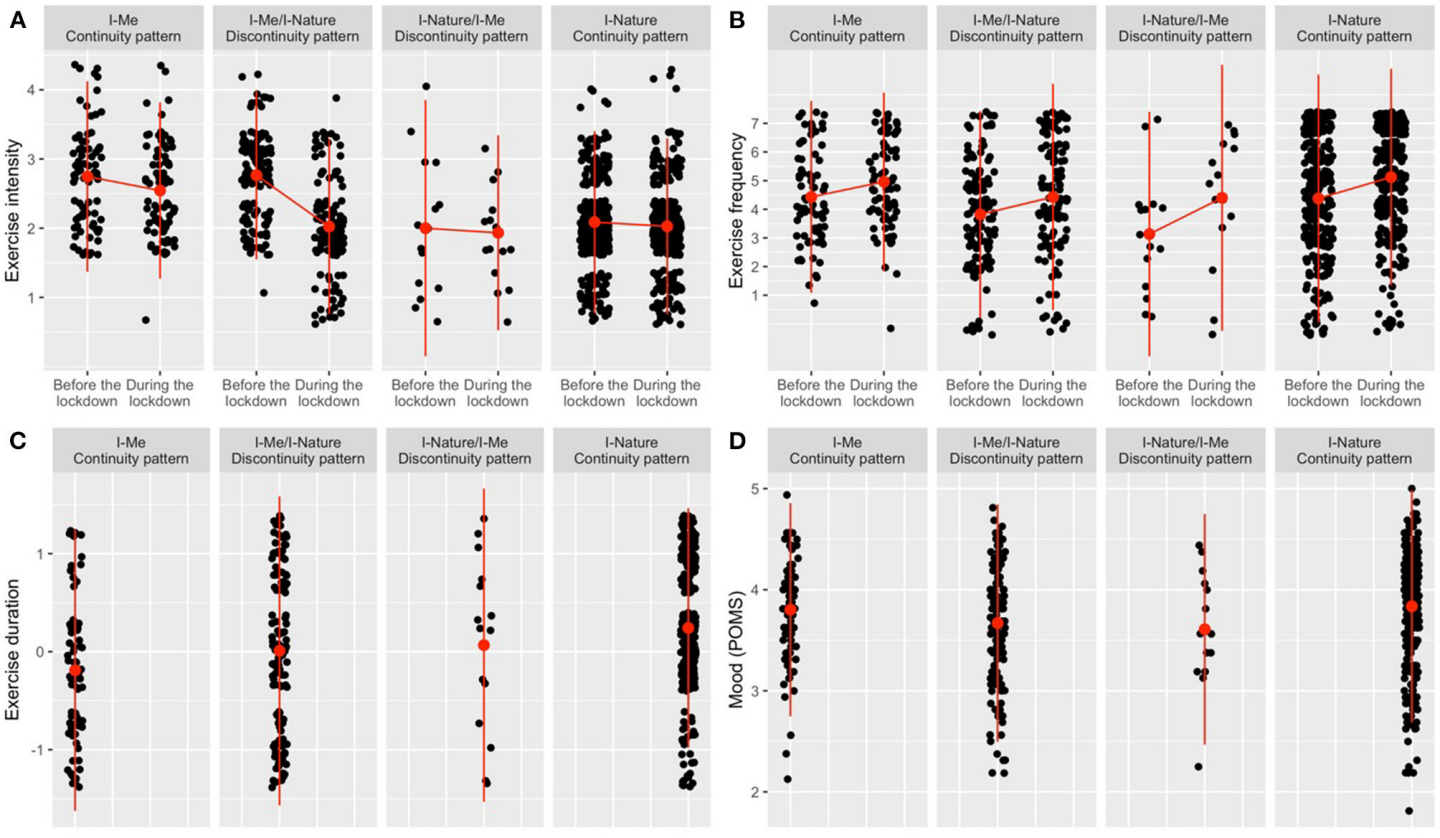
Changes in exercise Intensity **(A)**, Frequency **(B)**, Duration **(C)**, and Mood **(D)** in continuity/discontinuity patterns.

Means and standard deviations are given in Table 2. Differences and similarities in exercise intensity, frequency, and duration are illustrated in Figure 2.

**Table 2:** Means and standard deviations of exercise outcomes and mood in continuity/discontinuity pattern groups.

**Continuity/dis-continuity pattern**	**Time**	**n**	**Exercise frequency M (SD)**	**Exercise intensity M (SD)**	**Exercise duration^a^ M (SD)**	**Mood^b^ M (SD)**
I–Me/I–Me	Before	79	4.43 (1.68)	2.75 (0.69)		
	During	79	4.96 (1.56)	2.54 (0.64)	−0.19 (0.72)	3.80 (0.53)
I–Me/I–Nature	Before	128	3.82 (1.80)	2.77 (0.61)		
	During	128	4.43 (1.98)	2.02 (0.63)	0.01 (0.79)	3.67 (0.59)
I–Nature/I–Me	Before	15	3.13 (2.13)	2.00 (0.93)		
	During	15	4.40 (2.32)	1.93 (0.70)	0.07 (0.80)	3.61 (0.57)
I–Nature/I–Nature	Before	297	4.38 (2.16)	2.09 (0.66)		
	During	297	5.12 (1.89)	2.03 (0.64)	0.24 (0.62)	3.84 (0.57)

a *Exercise duration was measured as a difference score (less, similar or longer), negative values indicate less exercise during the lockdown*.

b *Mood was measured only once, during the lockdown*.

#### Intensity

Participants in the I–Me continuity pattern (*V* = 412.5, *p* = 0.008, *r* = 0.30; medium effect) and those in the I–Me/I–Nature discontinuity pattern (*V* = 3590, *p* < 0.001, *r* = 0.69; large effect) reported lower exercise intensity during the lockdown as compared with their exercise intensity before the lockdown. No significant changes were evident in the other patterns (Figure 2A).

#### Frequency

Exercise frequency was higher during the lockdown in all four groups, that is in the I–Me continuity pattern (*V* = 266, *p* = 0.006, *r* = 0.29; small effect), the I–Nature continuity pattern (*V* = 2983.5, *p* < 0.001, *r* = 0.40; moderate effect), and in the I–Me/I–Nature (*V* = 1706, *p* = 0.004, *r* = 0.23; small effect) and I–Nature/I–Me discontinuity patterns (*V* = 10.5, *p* = 0.049, *r* = 0.54; large effect) (Figure 2B).

#### Duration

Exercise duration was significantly different (small effect) in the four continuity/discontinuity patterns, H(3) = 25.9, *p* < 0.001, η^2^ = 0.04. *Post-hoc* multiple comparisons (Dunn-Bonferroni) revealed significant differences between the I–Me and the I–Nature continuity patterns (*z* = 4.75, *p* < 0.001), and between the I–Me/I–Nature discontinuity and the I–Nature continuity pattern (*z* = 2.96, *p* = 0.031) (Figure 2C).

### Differences in Mood Between Participants From the Continuity/Discontinuity Patterns

We found a significant (small effect) of “type-shift” on “mood,” *F*(3, 515) = 3.01, *p* = 0.030, η^2^ = 0.02. The *post hoc* tests indicated that this effect is caused by only one significant contrast. Those in the I–Me/I–Nature discontinuity pattern reported worse mood than those in the I–Nature continuity pattern (*q* = 0.17, *p* = 0.030). Calculated means and standard deviations are given in Table 2, and the data is illustrated in Figure 2D).

None of the ANCOVAs controlling for the potential covariate effects of exercise frequency, session duration, and intensity were significant. Detailed results of these tests are available upon request from the corresponding author.

## Discussion

Following the philosophical assertion that different types of exercise are related to different ways of “worldmaking,” this study sought to understand whether probable discontinuities in types of exercise participation following the COVID-19 pandemic manifest in the way people find themselves in the world as indicated by their mood. The main findings of this study were that (1) approximately one third of active Finnish adults changed their type of exercise/sport participation during the lockdown in the early weeks of the pandemic; (2) the most typical change was from I–Me to I–Nature-type activities; and (3) those who shifted from I–Me to I–Nature reported lower mood compared to those who remained in I–Nature. However, the effect on mood was small.

Our findings on the changes between exercise type categories following the lockdown are unsurprising. That is, when exercise facilities (e.g., gyms, swimming pools, sports halls) were closed and team sport activities put on hold, many people oriented themselves to outdoor activities, such as walking, jogging, and cycling. On average, our study participants from Finland were exercising more frequently during the lockdown than before the lockdown, which aligns with the findings of the international study (Brand et al., 2020). Previous studies indicate different patterns in how participation in physical activity and exercise has changed in different countries, which is probably related to various factors including the type of questionnaires used as well as how strict lockdown measures have been implemented. Notably, there were no restrictions on access to nature in Finland in April-May when we collected our data. At the same time, not having to commute to work may have provided extra time for exercising more frequently for many people.

The intensity of exercise decreased significantly for those who shifted from I–Me to I–Nature and for those who remained in I–Me. For example, those who had been used to exercising at a gym might have lacked the equipment or a motivating instructor to maintain the same intensity of exercise and replaced their previous exercise routines with less intense activities such as walking. However, for those who remained in the I–Nature category, and who assumedly had been mainly exercising without an instructor or equipment also before the pandemic, the intensity did not decrease. Interestingly, those who remained in I–Me reduced duration, those who remained in I-Nature increased it, and those who shifted the type of activity from I–Me to I–Nature reported no change. This indicates that there were also some changes in the two continuity patterns, as people were adapting to the new world under the pandemic. It is likely that a number of unobserved factors (e.g., type of work, family life, etc.) had an impact on the identified patterns.

Despite its small effect size, the observed effect on mood might be interesting. It has been shown in previous studies that forced abstinence from a chosen type of exercise can be associated with mood disturbances (Chan and Grossman, 1988; Szabo and Parkin, 2001). However, while Chan and Grossman's (1988) study included injured runners and Szabo and Parkin's (2001) participants (martial artists) were instructed not to participate in any strenuous physical activity for seven days, our study extends these findings by showing that even if participants in the I–Me/I–Nature discontinuity pattern exercised more frequently than before discontinuity, they still reported lower mood compared to those in the I–Nature continuity pattern. Therefore, not only the quantity, but also the quality of our involvement in exercise may matter in sustaining positive mood.

From an existential philosophical perspective that underpins Breivik's (2020) discussion of different ways of worldmaking through sport and exercise, it could be argued that it takes time to immerse oneself in a particular exercise/sport life-world before it feels one's “own.” Kretchmar (2000) argued that meaningful movement experiences often arise as a result of long-term practice and familiarity with the activity, so that the participants “have transcended fear, confusion, awkwardness, movement mechanics, and all other introductory aspects of an activity” (p. 23). If moods form the existential background to our lives and relate to whether things and experiences in the life-world matter to us (Ratcliffe, 2013), it could be that the new exercise routine (e.g., changing from weightlifting to jogging) often does *not* immediately make embodied sense, which then reflects in our mood. Our sense of “life is OK” is tacit and embodied: A runner experiences a different sense of vigor, soreness, and fatigue than a weightlifter, and these bodily cues normally tell us that life is “as it should be.” In a bodily sense, a certain level of intensity in our favorite exercise feels “at home” and familiar to us, and it can take us time to be “at home” in a different mode of physical activity. Furthermore, involvement in certain sport or exercise can be an important life project to participants also in non-elite levels and previous studies show that the inability to continue in this specific activity can have detrimental effects on well-being (Allen Collinson and Hockey, 2007; Ronkainen et al., 2014). Just as spending time in “favorite places” has been associated with psychological benefits (Korpela et al., 2010), “favorite exercise” might function as a symbolic place that we can go to and that gives us comfort and reinvigorates us. Future research could explore whether “favorite exercise” is a protective factor for maintaining a positive mood under pandemic or other disruptive experiences, as well as in life in general.

While the philosophical conceptualizations of “worldmaking” and mood as well as our findings on the negative impact of discontinuity on mood can open up a new perspective on understanding the meaning of exercise in people's lives, the effect sizes were small. Our findings must therefore be considered preliminary. One of the reasons for the small effect size may be that the way we find ourselves in the world is shaped by a variety of our involvements besides exercising. Furthermore, the psychological measure that we used in our empirical research might not optimally operationalize what is captured by the more philosophical concept of mood. With these cautions in mind, our finding provides an interesting hypothesis for future studies: The mode of exercise/sport people have chosen as their “own” could be a key exercise-related protective aspect that can support positive mood. If this findings was to be confirmed in future studies, this would indicate that it is important to help people to continue their preferred mode of exercise or sport participation. Simply keeping active in any means possible might not provide the same benefits to mood.

Finally, to broaden the discussion to recent debates on the role of exercise during COVID-19 pandemic, Malcolm and Velija (2020) highlighted that the public messages about the importance of staying active under lockdown have intensified people's feelings of guilt if they are not exercising. If people cannot engage in their preferred mode of exercise, they might choose another way of keeping active to avoid the negative feelings associated with inactivity. From the survey data, we are unable to infer our participants' intentions and whether part of their involvement in the new types of activities was a response to social pressure to keep active. However, we can speculate whether avoiding guilt could have contributed to lower mood in those participants who had to change their type of exercise from I–Me to I–Nature (even if being in nature has been reported to give psychological benefits overall; Van den Berg et al., 2010). Taken together, our finding highlights previous arguments that mood improvement is not an automatic reaction to exercise or sport participation, but seems to be a more complex process that concerns how we relate to exercise and sport and our world through being physically active.

### Limitations of the Study

The findings here should be interpreted with several limitations in mind. We were forced to utilize convenience sampling due to available resources and time limits for data collection. Consequently, our sample is not representative of the general population in that it mostly included well-educated participants who had a rather high-income status and who exercised or played sport 4–5 times per week. As Park and Kang (2008) noted, more educated individuals are more likely to exercise regularly. Smith et al. (2020) also reported that adults in the UK with lower income were less likely to be active during this pandemic-related lockdown. Although it was impossible to investigate this effect in our study, it is likely that for our participants their exercise and sport routines might be more important than for less active adults and be more strongly related to their mood. The season was changing during the data collection period, which could also have affected exercise and sport behavior. Furthermore, we were not able to collect mood data before the lockdown and therefore we do not have information on how participants' mood changed in response to the lockdown.

There can be several other factors that threaten mood during a lockdown. From this perspective, the small effect size of this finding is not very surprising, given that exercise behavior is only one aspect of daily life and one's identity. From the two discontinuity types analyzed, only one of them, the shift from I–Me to I–Nature, was significantly different from the I–Nature/I–Nature continuity pattern, suggesting that the type of discontinuity can matter, and this should be tested in larger, balanced samples. Any nonsignificant differences may be real, or due to lack of statistical power for these particular comparisons, which was not designed a priori. Furthermore, I–You and I–Society type of activities had an insufficient number of participants and were removed from the analysis. Therefore, these findings apply only to comparing changes between the categories we included in the final data and do not tell about other exercise type categories and the impact of (dis)continuities in them.

Finally, Breivik's (2020) fourfold framework is not a stable model but more like an exploration of ideal types of exercise/sport and their underlying “world relations.” Assigning certain activities such as jogging to either the I–Me or I–Nature category is certainly not clear cut. However, the main finding that changing your preferred type of exercise/sport is associated with different psychological outcomes compared to continuing a routine within the same type provides us with an indication that the type of exercise might matter in terms of our mood.

## Conclusions

From our findings, it could be suggested that people have their own mode of exercising or doing sport that is associated with their well- or ill-being. While researchers have reported an association between outdoor exercise in green spaces with subjective well-being (Pietilä et al., 2015), our research indicated that starting to exercise outdoors following lockdown was not associated with better mood. Perhaps being forced to disengage from the “favorite” activity (e.g., going to the gym) and the rupture of habits has a more negative influence on people's attunement to the world than outdoor activities can “repair.” While we cannot know the practical significance of the measured differences in mood variables between the groups in participants' everyday lives, the study provides an interesting hypothesis for future studies; that is, our engagement with a specific mode of exercise/sport, rather than just any exercise or physical activity available, can be important for our well-being. Given the COVID-19 pandemic is likely to result in further closures of exercise/sport facilities and disruption of group exercise and team sports also in the future, attempts to help people build some degree of continuity in their exercise/sport life-world is a practical recommendation stemming from the study.

## Data Availability Statement

The raw data supporting the conclusions of this article will be made available by the authors, without undue reservation.

## Ethics Statement

Ethical review and approval was not required for the study on human participants in accordance with the local legislation and institutional requirements. The participants provided their written informed consent to participate in this study.

## Author Contributions

NR framed the theoretical considerations for this study. NR and RB cooperatively developed the exact research question for the article. All authors (NR, AP, OT, and RB) made additional substantial, direct and intellectual contributions to finalization work, and approved it for publication.

## Conflict of Interest

The authors declare that the research was conducted in the absence of any commercial or financial relationships that could be construed as a potential conflict of interest.
