# Supplementary material for: Continuity and Discontinuity of Sport and Exercise Type During the COVID-19 Pandemic. An Exploratory Study of Effects on Mood
Source: Front Psychol. 2021 Feb 12;12:622876. doi: 10.3389/fpsyg.2021.622876 (PMC7907513; doi:10.3389/fpsyg.2021.622876)
Supplement: Supplementary file 1 [file Table_1.docx]

Supplementary Table 1. The categorisation of primary exercise types.

| **"I - Me"** | **"I - You"** | **"I - Society"** | **"I - Nature"** |
| --- | --- | --- | --- |
| Gym | Badminton | Basketball | Walking |
| Resistance training | Tennis | Soccer | Jogging/running |
| Home workout | Squash | Volleyball | Cycling |
| Yoga | Boxing | Ice Hockey | Walking the dog |
| Running on treadmill | Kickboxing | Floorball | Walking in the nature |
| Track running | Thai boxing | Futsal | Nordic walking |
| Walking on treadmill |  | Ringette | Orienteering |
| Workout |  | Ball games (team sports) | Endurance training |
| Cross training |  |  | Horse riding |
| Crossfit |  |  | Hiking |
| Circuit training |  |  | Cycling (to work) |
| Aqua running |  |  | Frisbeegolf |
| Aqua exercise |  |  | Nordic skating |
| Cycling (stationary) |  |  | Trail running |
| Kettlebell |  |  | Trekking |
| HIIT |  |  | Skiing (cross-country) |
| Group exercise class |  |  | Skiing (downhill) |
| FUSTRA |  |  | Skating |
| Everyday activity |  |  | Building a house |
| Climbing |  |  | Silviculture activities |
| Dance |  |  | Gardening |
| Gymnastic exercises |  |  |  |
| Pilates |  |  |  |
| Rehabilitation |  |  |  |
| Swimming |  |  |  |
